# NTRK1/TrkA Signaling in Neuroblastoma Cells Induces Nuclear Reorganization and Intra-Nuclear Aggregation of Lamin A/C

**DOI:** 10.3390/cancers13215293

**Published:** 2021-10-21

**Authors:** Lukas Funke, Thilo Bracht, Sebastian Oeck, Karin Schork, Markus Stepath, Sabine Dreesmann, Martin Eisenacher, Barbara Sitek, Alexander Schramm

**Affiliations:** 1 Medizinisches Proteom-Center, Medical Faculty, Ruhr-University Bochum, 44801 Bochum, Germany; lukas.funke@rub.de (L.F.); thilo.bracht@rub.de (T.B.); karin.schork@rub.de (K.S.); Markus.stepath@rub.de (M.S.); martin.eisenacher@rub.de (M.E.); barbara.sitek@rub.de (B.S.); 2 Department of Anesthesia, Intensive Care Medicine and Pain Therapy, University Hospital Knappschaftskrankenhaus Bochum, 44892 Bochum, Germany; 3 Medical Proteome Analysis, Center for Protein Diagnostics (ProDi), Ruhr-University Bochum, 44801 Bochum, Germany; 4 West German Cancer Center, Department of Medical Oncology, University Hospital Essen, University of Duisburg-Essen, 45147 Essen, Germany; sebastian.oeck@uk-essen.de (S.O.); sabine.dreesmann@uk-essen.de (S.D.)

**Keywords:** phosphoproteomics, mass spectrometry, phosphorylation, proteomics, MYCN, nuclear foci, TrkA, NTRK1, neuroblastoma

## Abstract

**Simple Summary:**

Neuroblastoma (NB) accounts for 15% of all cancer-related deaths of children. While the amplification of the Myc-N proto-oncogene (MYCN) is a major driver of aggressive NB, the expression of the neurotrophin receptor, NTRK1/TrkA, has been shown to be associated with an excellent outcome. MYCN downregulates NTRK1 expression, but it is unknown if the molecular effects of NTRK1 signaling also affect MYCN-induced networks. The aim of this study was to decipher NTRK1 signaling using an unbiased proteome and phosphoproteome approach. To this end, we realized inducible ectopic NTRK1 expression in a NB cell line with MYCN amplification and analyzed the proteomic changes upon NTRK1 activation in a time-dependent manner. In line with the phenotypes observed, NTRK1 activation induced markers of neuronal differentiation and cell cycle arrest. Most prominently, NTRK1 upregulated the expression and phosphorylation of the nuclear lamina component Lamin A/C. Moreover, NTRK1 signaling also induced the aggregation of LMNA within nucleic foci, which accompanies differentiation in other cell types.

**Abstract:**

(1) Background: Neuroblastomas (NBs) are the most common extracranial solid tumors of children. The amplification of the Myc-N proto-oncogene (MYCN) is a major driver of NB aggressiveness, while high expression of the neurotrophin receptor NTRK1/TrkA is associated with mild disease courses. The molecular effects of NTRK1 signaling in MYCN-amplified NB, however, are still poorly understood and require elucidation. (2) Methods: Inducible NTRK1 expression was realized in four NB cell lines with (IMR5, NGP) or without MYCN amplification (SKNAS, SH-SY5Y). Proteome and phosphoproteome dynamics upon NTRK1 activation by its ligand, NGF, were analyzed in a time-dependent manner in IMR5 cells. Target validation by immunofluorescence staining and automated image processing was performed using the three other NB cell lines. (3) Results: In total, 230 proteins and 134 single phosphorylated class I phosphosites were found to be significantly regulated upon NTRK1 activation. Among known NTRK1 targets, Stathmin and the neurosecretory protein VGF were recovered. Additionally, we observed the upregulation and phosphorylation of Lamin A/C (LMNA) that accumulated inside nuclear foci. (4) Conclusions: We provide a comprehensive picture of NTRK1-induced proteome and phosphoproteome dynamics. The phosphorylation of LMNA within nucleic aggregates was identified as a prominent feature of NTRK1 signaling independent of the MYCN status of NB cells.

## 1. Introduction

Neuroblastomas (NBs) are the most common extracranial solid tumors that occur almost exclusively in children. Most cases are diagnosed within the first year of life and only 10% of NBs are diagnosed after the age of five [1]. NBs comprise 6–10% of all childhood cancers and are responsible for 15% of all cancer-related deaths of children [1]. While the majority of NBs are found in the adrenal gland, the cell of origin for NB has not been uncovered. Recent advances in single-cell profiling suggested that more benign neuroblastomas resemble Schwann cell precursors [2]. However, a large study comparing neuroblastoma and normal fetal adrenal tissue revealed a pan-neuroblastoma pattern, suggesting a common precursor of human NB [3]. In mice, the ectopic expression of neuroblastoma oncogenes, MYCN and (anaplastic lymphoma kinase) ALK, in neural crest precursors was sufficient to drive tumorigenesis in a transplant model [4]. These findings are corroborated by genetic models, in which the neural crest-specific expression of MYCN and/or ALK gives rise to neuroblastic tumors in mice and zebrafish [5,6,7].

In the past, several genetic factors have been found to correlate with unfavorable outcome in neuroblastoma, including loss of heterozygosity at chromosomes 1p [8], trisomy of 17q [9] and the amplification of the N-myc proto-oncogene protein (MYCN) [10]. Additionally, mutations in the ALK domain are linked to rare familial cases as well as 8% of sporadic neuroblastoma [11,12]. However, it was demonstrated that the presence or absence of telomere maintenance is the major determinant of clinical outcome in NB [13]. Here, genomic rearrangements at the TERT locus or MYCN expression can trigger telomerase activation. On the contrary, less aggressive clinical courses have been linked to the expression of the neurotrophin receptors, NTRK1/TrkA. NTRK1 and its activation by nerve growth factor (NGF) have been shown to induce differentiation and increase the immunogenicity of human neuroblastoma cells [14,15]. Still, the interaction between features of aggressive neuroblastoma that downregulate NTRK1 signaling is less well understood. MYCN has been found to suppress NTRK1 transcription by interaction with a repressive complex at the NTRK1 promoter [16]. Transcriptional profiling revealed that the major antagonistic programs driving gene expression in neuroblastoma are linked to either NTRK1 or MYCN expression [17]. Proteomic analyses of NTRK1 signaling have identified several targets of interest that contribute to differentiation (e.g., Cbl-b) [18], but these results did not provide a link to MYCN.

**Figure 1:**
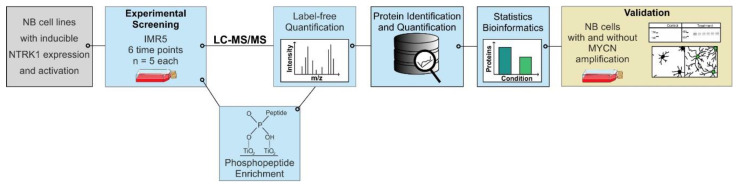
Schematic representation of the experimental workflow. For experimental screening, IMR5 cells underwent time course stimulation with NGF followed by proteome and phosphoproteome analysis, respectively. The generated data were statistically analyzed and selected proteins were further investigated in four NTRK1-expressing cell lines to validate the observations.

In the present study, we used four neuroblastoma cell lines with inducible NTRK1 expression to study dynamics in NTRK1 downstream signaling induced by NGF treatment. Using an MYCN-amplified cell line, IMR5, we monitored NTRK1-dependent changes in proteome and phosphoproteome composition using label-free LC-MS/MS analysis in a time-dependent manner (Figure 1). While several known NTRK1 downstream targets were identified, we also observed increased abundance and phosphorylation of Stathmin 1 (STMN1) and the nuclear lamina component Lamin A/C (LMNA). Using fluorescence microscopy, we demonstrated that NGF-induced NTRK1 signaling induces the accumulation of LMNA inside nuclear foci in all neuroblastoma cell lines that were analyzed. These data provide a comprehensive view on NTRK1-mediated signaling processes and point to a hitherto unknown NTRK1–LMNA axis in neuroblastoma, which could potentially explain the broad phenotypic changes and NTRK1-induced reprogramming of neuroblastoma cells.

## 2. Results

### 2.1. Validation of NTRK1/TrkA Expression and Activation in Four Established Human Neuroblastoma Cell Lines with Different MYCN Backgrounds

**Figure 2:**
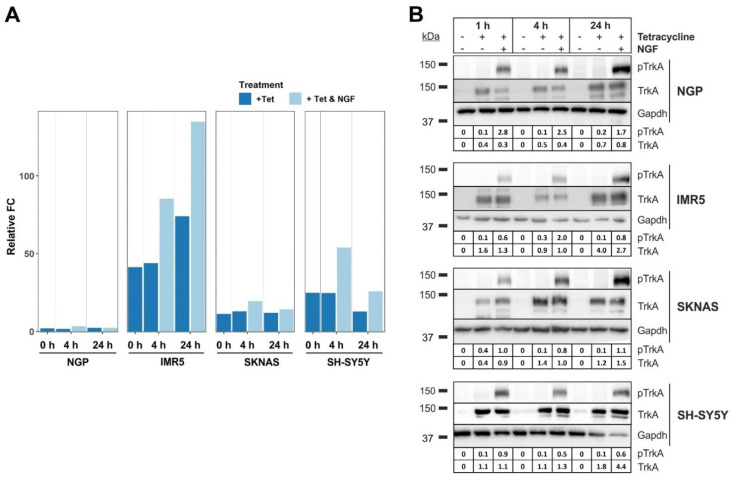
Validation of inducible expression and activation of NTRK1 in four neuroblastoma cell lines. (**A**) NTRK1 expression was verified in the NTRK1-negative cell lines NGP, IMR5, SKNAS and SH-SY5Y using qPCR at time points indicated. (**B**) NTRK1 activation was validated for time points t = 1 h, 4 h and 24 h upon treatment with NGF using immunostaining of phosphorylated NTRK1 (pTrkA Y674/675). Numbers indicate the intensity ratio of the TrkA/pTrkA bands and Gapdh. Uncropped blots were added in supplemental materials.

The inducible expression of NTRK1/TrkA and its activation via NGF were realized and validated in four established human neuroblastoma cell lines, NGP, IMR5, SKNAS and SH-SY5Y. The expression of NTRK1 was induced by the addition of tetracycline (Tet) to the medium and validated by qPCR (Figure 2A). The activation of NTRK1 by its ligand, NGF, was confirmed over a time course of 1 h, 4 h and 24 h using immunoblots (Figure 2B) and was most prominent after 24 h in all cell lines investigated. As we aimed to obtain hints on programs that are activated by NTRK1 in MYCN-overexpressing cells, we chose MYCN-amplified IMR5 cells for further analyses as they presented with the highest relative difference in NTRK1 expression (Figure 2A).

### 2.2. Proteome and Phosphoproteome Analyses Provide Time-Resolved Dynamics of NGF-NTRK1 Signaling in IMR5 Cells

In order to monitor time-course-dependent NTRK1 signaling, NTRK1 expression was induced in MYCN-amplified IMR5 cells. NTRK1 activation was again achieved by the addition of NGF and this was defined as t = 0. In addition, we incubated cells with NGF for 10 min, 1 h and 4 h to cover early signaling events. To account for proteome dynamics taking place with a certain latency, we additionally obtained samples after 12 h and 24 h after the addition of NGF (Figure S1). Upon protein extraction, samples (in biological quintuplicates) were subjected to proteome and phosphoproteome analyses using label-free liquid chromatography coupled to tandem mass spectrometry (LC-MS/MS). For phosphoproteome analysis, phosphorylated peptides were enriched using TiO_2_ affinity chromatography prior to analysis. For each time point, statistics were calculated in a pairwise manner between NGF-treated and NGF-untreated cells.

**Figure 3:**
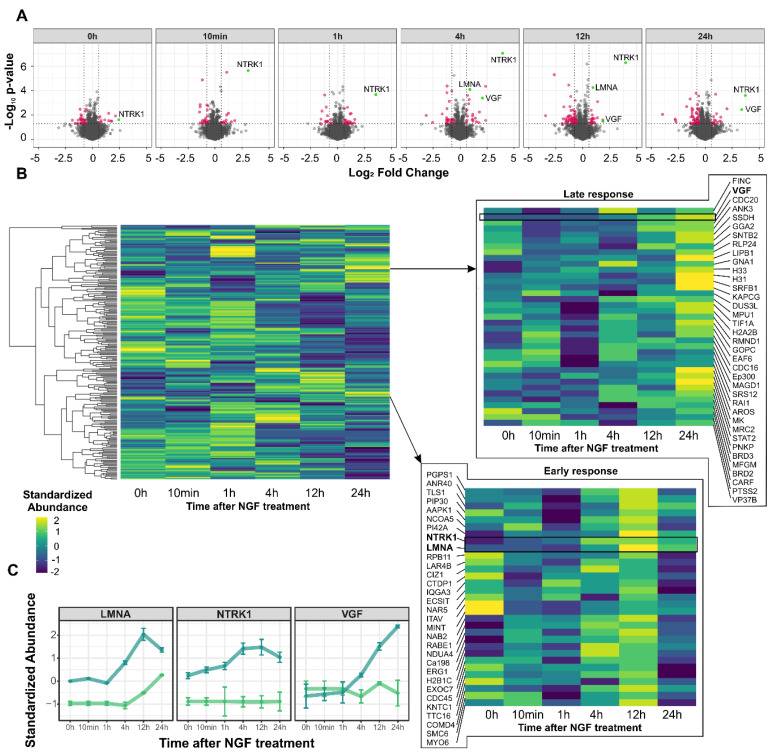
Proteome dynamics induced by NGF−mediated NTRK1 activation in IMR5 cells. (**A**) Volcano plots illustrating the differential expression of proteins for the time points indicated. Significantly differentially abundant proteins highlighted in red (*p*-value ≤ 0.05; FC ≤ −1.5 or ≥ 1.5), selected proteins highlighted in green. (**B**) Heatmaps representing hierarchical cluster analysis (Pearson correlation, complete linkage) of significantly regulated proteins. Magnifications show clusters of proteins with late and early increasing abundance, respectively. (**C**) Regulation profiles of selected protein candidates in NGF-treated cells (blue) compared to untreated controls (green).

In the proteome dataset, 3615 proteins were quantified (Table S1), of which 230 proteins passed the pre-defined filter criteria (*p*-value ≤ 0.05; FC ≤ −1.5 or ≥ 1.5; Table S2). Of those, 34 proteins were found to be differentially abundant already at baseline prior to NTRK1 activation (11 down- and 23 upregulated), thus indicating proteins that were affected by NTRK1 expression alone (Figure 3A). As a positive control, increased abundance of NTRK1 was observed in cells upon treatment with Tet. As soon as 1 h after NGF stimulation, the first modulations of protein abundancies were apparent, which resulted in 36 regulated proteins, of which 22 were down- and 14 upregulated. Major modulations of the proteome were observed after 4 h, 12 h and 24 h with 46, 78 and 61 differentially abundant proteins, respectively. Notably, we observed a tendency towards the downregulation of protein abundancies in NGF-treated compared to control cells. The abundance of the neurosecretory protein VGF (VGF) had been linked to NTRK1 activation in NB cells before [18]. Here, we found elevation of VGF expression 4 h upon NGF-mediated activation of NTRK1, confirming these results. Surprisingly, NTRK1 itself also showed an increasing abundance peaking at the 4 h and 12 h time points (Figure 3C). Cluster analyses of significantly regulated proteins revealed sets of proteins with common regulation profiles (Figure 3B). Here, proteins with a constant response towards NTRK1 activation were of particular interest, and amongst these were VGF and the nuclear laminar component Lamin A/C (LMNA).

**Figure 4:**
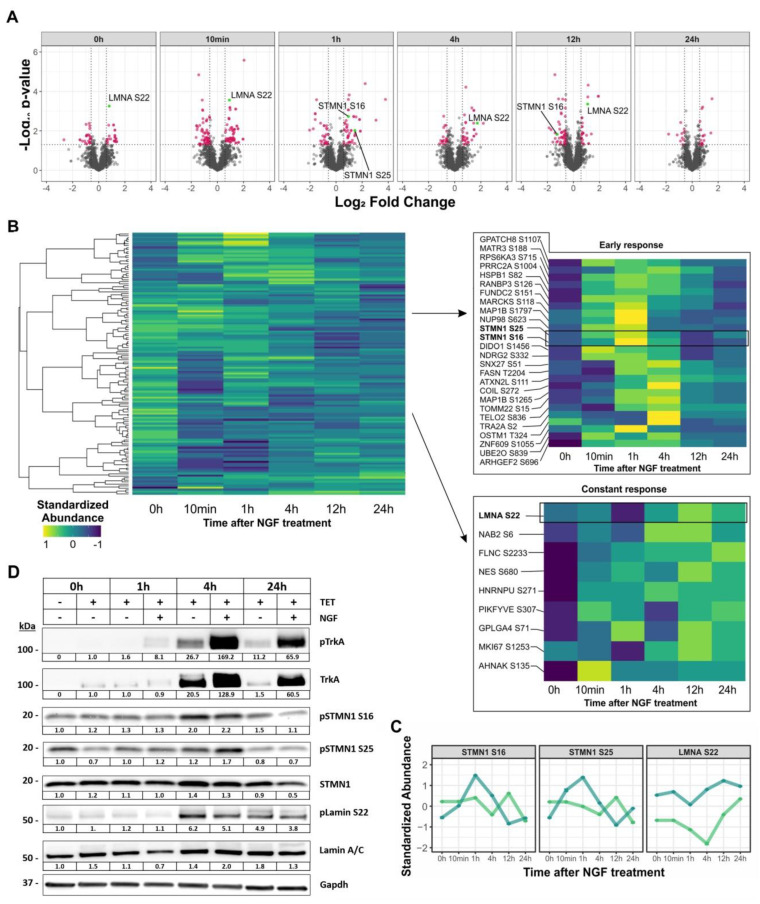
Phosphorylation dynamics induced by NGF−mediated NTRK1 activation in IMR5 cells. (**A**) Volcano plots representing the results of differential phosphoproteome analysis for the time points indicated. Significantly regulated p-sites are highlighted in red (*p*-value ≤ 0.05; FC ≤ −1.5 or ≥ 1.5) and prominently regulated p-sites are highlighted in green. (**B**) Heatmaps representing hierarchical cluster analysis (Pearson correlation, complete linkage) of significantly regulated p-sites. Magnifications show clusters of p-sites with early and constantly increasing abundance, respectively. (**C**) Regulation profiles of selected p-sites in NGF-treated cells (blue) compared to untreated controls (green). (**D**) Orthogonal validation of selected phosphorylation target sites shown in (**C**) using Western blot analyses of independent experiments. Cells were either treated with TET or a combination of TET/NGF for time points indicated to induce and activate NTRK1/TrkA, respectively. Phosphorylated states of NTRK1/TrkA, STMN1 and Lamin A/C and their regulation in relation to total protein expression are shown. Gapdh served as a loading control. Numbers indicate fold expression changes over time normalized to t = 0 h for each individual protein analyzed. Uncropped blots were added in Supplementary Materials.

For the phosphoproteome, 6982 phosphorylation sites (p-sites) were quantified (Table S3), of which 2843 were singly phosphorylated and scored as class I p-sites. Of these, 147 significantly regulated p-sites were further analyzed (Table S4). As for the entire proteome, regulated p-sites between NTRK1-expressing and control cells were observed in the absence of NGF treatment (Figure 4A,B). We did not identify differential p-sites for NTRK1, probably due to the known under-representation of tyrosine phosphorylation upon TiO_2_ enrichment. However, Western blot analysis did not suggest significant NTRK1 auto-phosphorylation in IMR5 cells (Figure 2B). NTRK1 activation by NGF led to a significant increase in phosphorylation dynamics peaking after 10 min with 56 regulated abundant p-sites, notably with a striking tendency towards lower abundance in the treatment group. For the 1 h, 4 h and 12 h time points, 34, 21 and 42 differential p-sites were observed. Only 14 proteins showed differential phosphorylation 24 h after NTRK1 activation by NGF. Clustering of differentially represented p-sites was performed to identify patterns of dynamic changes. Prominently, LMNA-pS22 was found to be more abundant in the treatment group compared to the control group and was significantly upregulated after 10 min, 4 h and 12 h (Figure 4C). Additionally, Stathmin (STMN1) p-S16 and p-S22 peaked 1 h after NTRK1 activation (Figure 4C). Both phosphorylation patterns and kinetics for these phosphosites could be orthogonally validated by Western blotting using phosphor-specific antibodies to LMNA-pS22, STMN1 p-S16 and p-S22 in all four cell lines and independent experiments (Figure 4D and Figure S2). Interestingly, we also observed an uncharacterized phosphoserine at MYCN S156 to be downregulated after 10 min; however, the lack of information or antibodies did not allow us to functionally follow up on this site.

### 2.3. NTRK1/TrkA Activation Modulates Cellular Pathways Related to Nuclear Lamina Integrity

**Figure 5:**
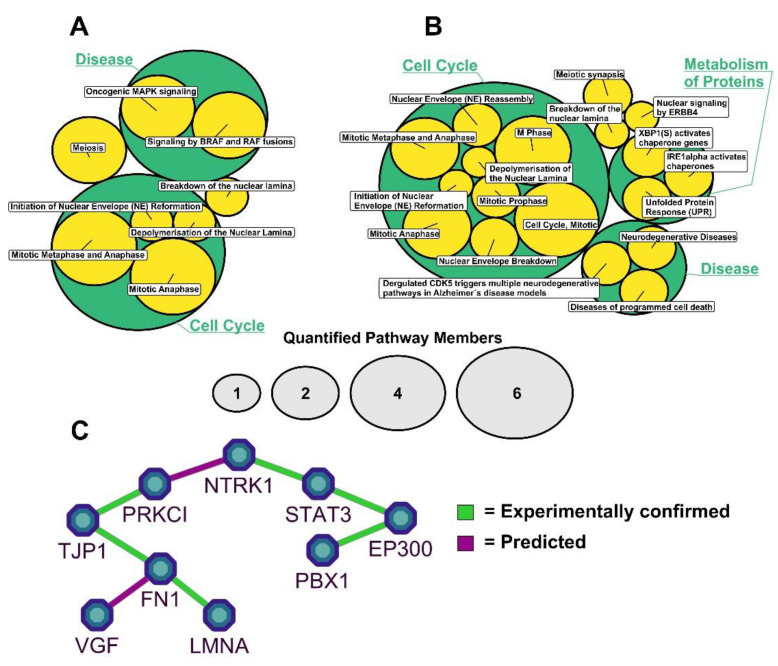
Pathway and network analysis of regulated proteins and phosphosites. Significantly differentially abundant proteins (**A**) and p-sites (**B**) were annotated with and analyzed for enrichment of regulated Reactome pathways. Bubbles illustrate the hierarchical nature of biological pathways and respective sub-pathways; bubble sizes indicate numbers of proteins/p-sites annotated with the respective pathway; only LMNA-containing pathways are displayed. (**C**) STRING protein interaction network illustrating interactors of NTRK1/TrkA and their functional connection to LMNA.

To further characterize biological processes and cellular pathways, which were modulated by the activation of NTRK1, proteins were annotated using Reactome pathways and enrichment analysis for significantly regulated proteins and p-sites were performed (Table S5). For the proteome, an enrichment of proteins related to cell cycle control was apparent. However, a striking enrichment of ontologies regarding the stability of the nuclear lamina was also evident (Figure 5A). Besides LMNA, the nuclear pore complex protein Nup98-Nup96 (NUP98) was annotated with the respective ontologies. Similar observations were made for the NTRK1-regulated phosphoproteome. Here, besides enriched categories regarding cell cycle control and protein turnover, again the enrichment of cellular processes related to nuclear lamina integrity was apparent (Figure 5B). STRING analysis resulted in a hypothetical functional network connecting NTRK1 with LMNA and VGF via Protein Kinase C (PRKCI) (Figure 5C).

### 2.4. NTRK1/TrkA Signaling Leads to LMNA Translocation and Aggregation in Nuclear Foci

**Figure 6:**
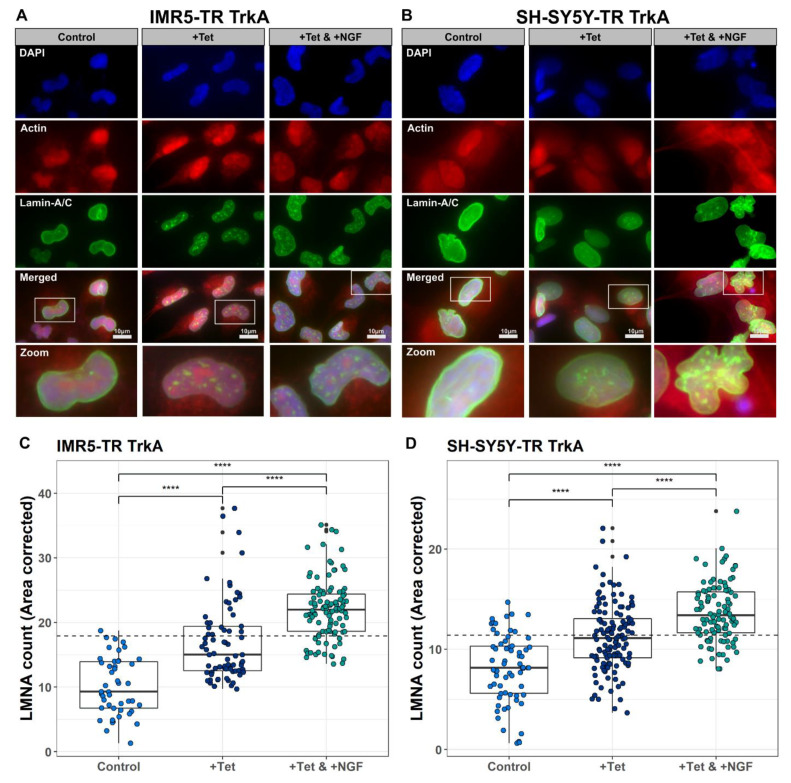
Changes in nuclear localization of LMNA upon NTRK1 expression and activation. Representative immunofluorescence stainings of LMNA (green) illustrating accumulation of LMNA within foci inside the nuclei upon NTRK1 expression and activation for IMR5 (**A**) and SH-SY5Y cells (**B**) (actin = red, DAPI = blue). In the merged pictures, zoom-ins are marked to highlight the foci formation in individual cells. (**C**,**D**) Area-corrected count of LMNA foci in IMR5 (**C**) and SH-SY5Y cells (**D**). Individual data points correspond to analyzed pictures. Significance (ANOVA with subsequent Tukey’s post hoc analysis **** p < 0.0005, *p*-values Bonferroni-corrected) is indicated by asterisks.

The results of the functional enrichment analyses directed our interest towards the interplay of NTRK1 signaling and the nuclear lamina. Hence, we investigated LMNA expression and localization using immune fluorescence imaging in the four available cell lines. In order to cover possible effects on neuronal differentiation processes, which are also controlled by NTRK1 signaling, we investigated the cell lines after a treatment time of two weeks. In the untreated control cells, LMNA was part of the nuclear lamina as expected (Figure 6 and Figure S2). In NTRK1-expressing cells, however, we observed a translocation of LMNA to the nucleus where it was localized within intra-nuclear aggregates in the four neuroblastoma cell lines analyzed: IMR5, SH-SY5Y, NGP and SKNAS (Figure 6 and Figure S3). We used automatic image analysis to count LMNA foci and found a highly significant difference between NTRK1-expressing and control cells (*p*-value < 0.0001). Upon treatment with NGF and the activation of NTRK1, SKNAS cells had reduced proliferation rates so that effects on LMNA distribution proved to be less feasible (Figure S3). In the other three cell lines, however, LMNA foci formation and changes in nuclear localization were even more pronounced (*p*-value < 0.0001), highlighting the functional relation between NTRK1 signaling and the increased number of LMNA foci.

## 3. Discussion

NTRK1/TrkA expression and MYCN amplification are hallmarks of excellent and dismal outcome, respectively, in childhood neuroblastomas (NBs). On the molecular level, NTRK1 confers pro-differentiation programs [19], increases the immunogenicity of NB cells [15] and enhances the proliferation of stromal cells [20]. On the contrary, MYCN drives NB aggressiveness by inducing proliferation, angiogenesis and by downregulating NTRK1 [16]. The aim of this study was to achieve a better understanding of the effects of NTRK1 activation on NB cells using in vitro models with and without amplification of the MYCN oncogene. This eventually would allow the identification of target proteins that are at the crossroads of NTRK1 and MYCN signaling. For this purpose, we used four neuroblastoma cell lines with inducible NTRK1 expression and activation, two of which were harboring MYCN amplification (IMR5, NGP), while the other two had normal MYCN copy numbers (SH-SY5Y, SKNAS, Figure 2). The impact of NTRK1 activation on the proteome and the phosphoproteome was delineated using the MYCN-amplified neuroblastoma cell line, IMR5. Proteins and their phosphosites were quantified using mass spectrometry and their regulation was evaluated in a pairwise manner for individual time points between 10 min and 24 h after NTRK1 activation. Overall, we quantified 3615 proteins and 2843 single phosphorylated class I p-sites, respectively, of which 230 proteins and 147 p-sites passed the filter criteria (Figure 3 and Figure 4). The results obtained here are on par with a comparable study by Emdal et al., who analyzed the NTRK1-regulated proteome in SH-SY5Y neuroblastoma cells [18]. Both studies are technically complimentary as Emdal et al. used SILAC labeling, whereas we performed label-free quantification, which has now been used more frequently in phosphoproteomics as it circumvents drawbacks of label-based approaches such as sample limitations [21]. Furthermore, label-free approaches proved to be beneficial in large-scale analyses, especially when multiple variables including time, receptor expression and receptor activation must be considered [22]. Interestingly, the number of significantly regulated proteins identified in this study exceeded those reported in Emdal et al. [18], while the latter reported a higher number of phosphopeptides. Among the commonly identified proteins in both studies that presented with altered phosphorylation levels upon NTRK1 activation were the neurosecretory protein VGF and Stathmin (STMN1). STMN1 regulates the cell cytoskeleton by destabilizing microtubules and is essential for cell cycle control [23]. Two major phosphorylation sites in STMN1 could be recovered in our study, STMN1-pS16 and STMN1-pS25. The phosphorylation of STMN1 at S16 is reported to abrogate tubulin binding capacity and thus reduces mitotic activity [24], while STMN1-pS25 is thought to play a role in malignant hematopoiesis [25]. Interestingly, STMN1-pS25 is known to be phosphorylated by PKCb1 downstream of NTRK1, and NGF reportedly induces STMN1 expression [26]. Another possible mediator of STMN1 phosphorylation is CAMK2a, which was previously described to be regulated in response to NGF exposure [27]. In neuroblastoma, STMN1 phosphorylation was reported to impact on metastasis by altering the levels of tyrosine-protein phosphatase non-receptor type 14 (PTPN14) [28] or in a tubulin-independent manner to enhance transendothelial migration via RhoA/ROCK signaling [29].

It is noteworthy that a differential abundance of phosphosites was frequently observed at early time points upon NTRK1 activation, while total protein levels were mostly altered at later time points (Figure 4 and Figure 5). However, some proteins also showed consistent phosphosite modulation at later time points. Importantly, we observed an increased abundance and phosphorylation of the nuclear lamina component Lamin A/C (LMNA) at S22 upon NTRK1 activation. These results were verified in IMR5 and three additional cell lines, SH-SY5Y, SKNAS and NGP (Figure 4D and Figure S2). However, effects were more pronounced in the MYCN-amplified IMR5 and NGP cells compared to SH-SY5Y and SKNAS cells with normal MYCN levels. Pathway analysis also suggested a correlation of NTRK1 activation with proteins involved in the stability of the nuclear lamina (Figure 5). A recent study linked LMNA to the differentiation of neuroblastoma cells in vitro, as the knockdown of LMNA enforced the upregulation of genes involved in self-renewal and stemness [30]. Furthermore, the hypermethylation of the LMNA promoter region in MYCN-amplified cells was suggested as a mechanism to downregulate LMNA [31,32]. Phenotypic analyses of the IMR5 cell line were conducted to investigate changes in cell structure, DNA and LMNA localization and structure as a consequence of prolonged NTRK1 expression and activation. Using immunofluorescence staining and automatic image analysis, we demonstrated that the activation of NTRK1 induces the nuclear relocation of LMNA and its accumulation within nuclear foci in IMR5 and SH-SY5Y cells (Figure 6, NGP and SKNAS in Figure S4). Disruption of the nuclear lamina alters the distribution of replication factors inside the nucleus and results in an inhibited DNA replication. It was shown that even LMNA distribution along the nuclear lamina was required to proceed from the initiation to the elongation phase of DNA replication [33]. Both NTRK1 activation in neuroblastoma cells and the induction of LMNA have been independently linked to differentiation. The results of our phosphoproteomic study and the phenotypic analyses now provide the first clues that NTRK1 signaling directly affects LMNA functions. Mechanistically, the disruption of the nuclear lamina alters the distribution of replication factors inside the nucleus and results in the inhibition of DNA replication. It was shown that a homogeneous LMNA distribution throughout the nuclear lamina was required to proceed from the initiation to the elongation phase of DNA replication and that this process critically depends on LMNA-S22 phosphorylation [34]. Further, Lamin A phosphorylation on Serine 22 is required for CDK1-catalysed nuclear envelope disassembly, and we could previously show that CDK1 is upregulated in aggressive neuroblastoma independently of MYCN status [35]. While the data presented here indicate that NTRK1 expression and activation also interfere with LMNA-S22 phosphorylation and nuclear lamina organization, it will require further work to dissect the impact of kinase-mediated LMNA phosphorylation on replication in neuroblastoma cells.

In summary, we here extend the current knowledge on NTRK1-induced proteins and processes to the level of effector proteins by proteomic and phosphoproteomic analyses. Uncovering the differential phosphorylation of LMNA and STMN1 upon NTRK1 activation gives new clues on NTRK1-mediated regulation of the differentiation and proliferation of neuroblastoma cells. Further studies should reveal the interdependence of LMNA, STMN1, TrkA and MYCN and their suitability for targeted intervention, from which patients with MYCN-amplified high-risk NB would ultimately benefit in the future.

## 4. Materials and Methods

### 4.1. Cell Culture and Cell Lines

Inducible expression of NTRK1/TrkA was established in four human neuroblastoma cell lines, two of which were MYCN-amplified (NGP, IMR5) and two were MYCN-single copy (SKNAS and SH-SY5Y).

To utilize a selective expression of NTRK1, a two-vector system of a Tet-repressor and a Tet-responsive element was used. The cell lines were transfected with pcDNA6/TR, containing a tetracycline repressor gene and pT-REx-DEST30 (Invitrogen, Carlsbad, CA, USA) involving NTRK1 cDNA. By utilizing a medium containing blasticidine and G418 (ThermoFisher/Invitrogen), the selection of single cell clones could be performed [36]. Each of the transfected cell lines was suffixed with “TR-TrkA” to allow identification in comparison to the parental cell lines. NTRK1 expression was accomplished by addition of 1 μg tetracycline hydrochloride (HP63.1, Carl Roth, Karlsruhe, Germany) per ml medium. Authentication of all used cell lines before and after transfection was performed via STR genotyping. All reagents used for cell culture were obtained from Gibco (Carlsbad, CA, USA). PCR with Mycoplasma-specific primers (IDT) was utilized to regularly check for possible contamination of *Mycoplasma* sp. in the cultivated cells.

### 4.2. Western Blot Analyses

Twenty-microgram proteins were separated using 4–20% CRITERION® TGX Stain-Free™ Gels (300 V, 20 min) and subsequently transferred to Trans-Blot® Turbo™ Nitrocellulose membranes (both Bio-Rad, Hercules, CA, USA). The membranes were blocked for 1 h at RT with 5% BSA in TBST (0.1% Tween20), before the primary antibodies were applied diluted 1:1000 in the same buffer (TrkA, Cell Signaling [2510]; pTrkA (Y674/675), Cell Signaling [4621S]; LMNA, Abcam [ab108595]; pLMNA (S22), Abcam [ab138450]; STMN1, abcam [ab52630]; pSTMN1 (S16), Abcam [ab47328]; pSTMN1 (S25), Abcam [ab194752]), except anti-Gapdh (EMD Millipore [MAB374]), which was diluted 1:2000. The membranes were incubated over night at 4 °C, washed with TBST and consecutively incubated with the appropriate secondary antibodies (anti-Rabbit IgG HRP, Thermo Scientific [31460]; anti-Mouse IgG HRP, Thermo Scientific [31430]) for 1 h at RT. Bands were visualized with SuperSignalTM Chemiluminescent Substrate (Thermo Fisher Scientific, Waltham, MA, USA) and documented using a CHEMI SMART 5000 System (Vilber Lourmat, Eberhardzell, Germany).

### 4.3. Activation of Ectopically Expressed NTRK1/TrkA and Preparation of Lysates for Proteome Analyses

NTRK1/TrkA expression was induced by tetracycline for 24 h before TrkA was activated by addition of NGF (100 ng/mL, R&D Systems, Minneapolis, NE, USA). The control group was left untreated. Cells were harvested 0 h, 10 min, 1 h, 4 h, 12 h and 24 h after NGF treatment. To account for different culture times, adjusted cell numbers were seeded to warrant homogeneous densities at the time of harvesting (1.3 × 10^5^ cells/mL were seeded for 0–4 h time points, 1 × 10^5^ cells/mL for 12 h and 7.5 × 10^4^ cells/mL for 24 h). Cells were washed three times with ice-cold PBS and subsequently incubated with 500 µL RIPA buffer containing PhosStop and cOmplete phosphatase and protease inhibitors (both Roche, Basel, Switzerland) and 0.5 µL Benzonase (Merck, Darmstadt, Germany) for 30 min on ice. Cells were harvested and centrifuged at 16,000× *g* for 20 min (4 °C).

Supernatants were collected and NTRK1 activation was confirmed by Western blot and qPCR according to protocols described in [14,15]. Protein precipitation via acetone (−20 °C) was performed overnight and the resulting pellets were resolved in urea buffer (30 mM Tris HCl pH 8.5, 7 M Urea, 2 M Thiourea). The resulting protein concentrations were checked via Bradford assay (Bio-Rad, Hercules, CA, USA). Subsequently, tryptic digestion of 1mg protein per sample was performed. DTT was added to a final concentration of 5 mM for 30 min at 60 °C, and the reaction was stopped with iodoacetic acid (final conc. 15 mM) for 30 min at RT in the dark. After that, the samples were diluted 1:7 in 50 mM ammonium bicarbonate and a tryptic digestion at 37 °C was performed overnight (trypsin 1:50 ratio, SERVA, Heidelberg, Germany). Digestion was stopped by addition of 0.5% TFA and a subsequent peptide purification was performed using Oasis HLB PLUS cartridges (Waters, Eschborn, Germany). The resulting peptide concentrations were determined utilizing amino acid analysis on an ACQUITY-UPLC (Waters GmbH, Eschborn, Germany) as previously described [37].

### 4.4. Phosphopeptide Enrichment

Phosphopeptide enrichment was performed as described previously [38,39]. Briefly, 100 µg of peptides per sample was mixed with 1 mL of loading buffer (80% ACN, 5% TFA, 1 M glycolic acid) and applied to 0.6 mg of TiO_2_-beads (Titansphere, 5 μm, GL Science, Tokyo, Japan). The peptides were allowed to bind to the beads and washed with 200 μL loading buffer (200 μL 80% ACN, 1% TFA and 200 µL 20% ACN, 0.5% TFA). Subsequently, the beads were vacuum-dried, dissolved in 50 μL 1% ammonium hydroxide (pH 11.3) and incubated for 10 min at 4 °C. Beads were removed by centrifugation using self-packed C8 stage tips (Empore SPE C8, Sigma-Aldrich, St. Louis, MO, USA). Eluates were vacuum-dried and dissolved in 0.5% ACN, 0.5% acetic acid before they were purified using self-packed C18 stage tips (Empore SPE C18, Sigma-Aldrich, St. Louis, MO, USA) and eluted with 50% ACN, 0.5% acetic acid. The samples were stored, vacuum-dried and re-suspended in 0.1% TFA before LC-MS/MS analysis.

### 4.5. LC-MS/MS Analysis

All LC-MS/MS analyses were executed on an Ultimate 3000 RSLCnano HPLC (Dionex, Idstein, Germany), which was coupled to a Q Exactive HF instrument (Thermo Fisher Scientific, Waltham, MA, USA). The measurements were carried out in random order. Of all samples, 300 ng of tryptic peptides was applied per measurement except for phosphoproteome analysis, where the entire eluted sample was used. Sample pre-concentration was achieved on a C18 trap column (Acclaim PepMap 100; 100 μm × 2 cm, 5 μm, 100 Å) in a period of 7 min at a flow rate of 30 μL/min using 0.1% TFA, and peptides were then transferred to a Nano Viper C18 analytical column (Acclaim PepMap RSLC; 75 μm × 50 cm, 2 μm, 100 Å). Separation of peptides was performed by applying a gradient from 5–40% solvent B over 120 min at 400 nL/min and 60 °C (solvent A: 0.1% formic acid (FA); solvent B: 0.1% FA, 84% ACN). For all samples, full-scan mass spectra were acquired in the Orbitrap analyzer in profile mode at a resolution of 60,000 at 400 *m*/*z* and within a mass range of 350–1400 *m*/*z*. Data-dependent mode at a resolution of 30,000 was utilized. The 10 most abundant ions per scan were selected for the MS/MS measurements and fragmented with higher-energy collision-induced dissociation (HCD; normalized collision energy (NCE) = 28).

### 4.6. Mass Spectrometry Data Analysis

Data analysis was performed using MaxQuant (ver. 1.6.5.0). If not specified otherwise, default settings were used. Static modification was set for cysteine (carbamidomethyl). Dynamic modifications were considered for methionine (oxidation) and phosphorylation of serine, threonine and tyrosine residues. Spectra were searched against the UniprotKB/SwissProt database (release 2019_08; 560,823 entries) restricted to *Homo sapiens.* The “match between runs” feature was enabled for both proteomics and phosphoproteomics analyses, as suggested in recent benchmark studies [39,40]. The label-free quantification algorithm (LFQ) was applied for proteome data, but not for phosphoproteome data. Only proteins quantified in at least 50% of the replicates in at least one experimental condition were considered for further analyses. Raw abundancies were subsequently normalized using the cyclic LOESS approach. Phosphorylation sites were included in subsequent analyses only when localization probability exceeded 75% (class I p-sites). The mass spectrometry proteomics data have been deposited at the ProteomeXchange consortium via the PRIDE partner repository with the dataset identifiers PXD026876 (proteome) and PXD026913 (phosphoproteome).

### 4.7. Statistical Analysis

Statistical procedures were conducted using R (r-project.org). For proteome data, t-tests between control and treatment groups were calculated based on Log_2_-transformed data for each of the analyzed time points (unpaired, two-sided). For phosphoproteome data, a Welch t-test was used accordingly. Fold changes (FC) were calculated as differences of mean Log_2_ abundancies, which were subsequently transformed to linear scale. The applied significance threshold was set to a *p*-value ≤ 0.05 and a fold change ≥1.5 or ≤−1.5. For proteome analysis, only proteins represented by at least two unique peptides were considered.

### 4.8. Pathway Analysis

Annotation with Reactome pathways and statistical enrichment analysis were performed using the R-package ReactomePA (v.1.34.0 [41]). Pathways with a *p*-value < 0.05 were considered to be significantly enriched and visualized using the R-package CirclepackeR (v.0.0.0.9). The results of STRING (v.11; [42]) network analyses were visualized using Cytoscape (v.3.7.2; [43]).

### 4.9. Immunofluorescence Staining and Quantitative Image Analysis

A total of 300 cells per chamber were seeded in 8-well chamber slides (Millicell EZ Slide, Merck) and incubated for 24 h before the respective experimental replicates were treated with Tet. After another 24 h. NGF was added and the cells were grown for 14 days. Cells were fixed with 3% PFA, 0.5% Triton-X100, 8% Sucrose followed by ice-cold methanol. Blocking was performed overnight at 4 °C using 5% FBS, 5% NGS, 0.2% Triton X-100 in PBS. Primary antibody staining was performed for 30 min at RT with anti-Lamin A/C rabbit (1:250, ab108595, Abcam, Cambridge, UK) in blocking solution. First, antibodies were visualized using anti-mouse AFplus 488 secondary antibodies (1:400, A32723, Thermo Fisher Scientific, Waltham, MA, USA) in blocking solution for 75 min. Subsequently, DNA and actin were visualized using DAPI/Hoechst mix (1:200, Thermo Fisher Scientific, Waltham, MA, USA) and Phalloidin-TRITC (1:40, P1951, Sigma-Aldrich, St. Louis, MO, USA) for 45 min. Documentation was performed with an Axio Cam MRM (Carl Zeiss Microscopy, Jena, Germany) and a 63×/1.4 Oil DIC M27 Plan-Apochromat Objective (Carl Zeiss Microscopy). The pictures were automatically processed with ImageJ (v.1.51j8). Area-corrected counting of LMNA accumulation within foci inside the nuclei upon NTRK1 expression and activation was performed utilizing the Focinator [44] tool (v.2.31). The Focinator was modified to exclude counting of LMNA accumulation in the nuclear membrane itself.

## 5. Conclusions

MYCN and NTRK1 exert opposing functions in neuroblastoma (NB). While MYCN promotes cell cycle progression and other features of aggressive tumor growth, NTRK1 induces differentiation and is associated with excellent patient outcome. In cell lines derived from human NB, the effects of MYCN are best studied and it is known that MYCN downregulates NTRK1 expression on a transcriptional level. Conversely, the effects of NTRK1 on MYCN-induced programs are less well understood. Here, we obtained the proteomic profiles of an MYCN-amplified neuroblastoma cell line upon ectopic NTRK1 expression and activation. This allowed us to identify several effector genes that are induced by NTRK, including known targets such as Stathmin (STMN1) and VGF. Of note, we uncovered a previously undescribed upregulation and subsequently altered subcellular distribution of Lamin A/C as a consequence of NTRK1 activation, which is also seen in MYCN-normal cells. Lamin A/C has been reported to correlate with the differentiation of normal and malignant cells. It will be interesting to study the function of Lamin A/C to further disentangle MYCN and NTRK1-regulated circuits, which are at the crossroads between proliferation and differentiation in neuroblastoma cells.

## Data Availability

The data presented in this study are available on request from the corresponding author (alexander.schramm@uk-essen.de). The mass spectrometry proteomics data have been deposited at the ProteomeXchange consortium via the PRIDE partner repository with the dataset identifiers PXD026876 (proteome) and PXD026913 (phosphoproteome).

## Supplementary Materials

The following are available online at https://www.mdpi.com/article/10.3390/cancers13215293/s1, Table S1: LFQ-normalized protein abundancies, Table S2: Significantly differentially abundant proteins, Table S3: CycLoess-normalized phosphosite abundancies, Table S4: Significantly differentially abundant phosphosites, Table S5: Results of Reactome pathway analysis, Figure S1: Validation of NTRK1/TrkA expression and activation in IMR5 control and treatment used for subsequent proteomics analyses, Figure S2: Validation of expression and activation patterns via pLMNA S22, pSTMN1 S16 and S25 in SY5Y, NGP and SKNAS, Figure S3: Changes in nuclear localization of LMNA upon NTRK1 expression and activation. Uncropped blots was added in supplemental materials.
