# Supplementary material for: NTRK1/TrkA Signaling in Neuroblastoma Cells Induces Nuclear Reorganization and Intra-Nuclear Aggregation of Lamin A/C
Source: Cancers (Basel). 2021 Oct 21;13(21):5293. doi: 10.3390/cancers13215293 (PMC8582546; doi:10.3390/cancers13215293)
Supplement: Supplementary file 1 [file cancers-13-05293-s001.zip › cancers-1433337-supplementary/cancers-1433337-non.pdf]

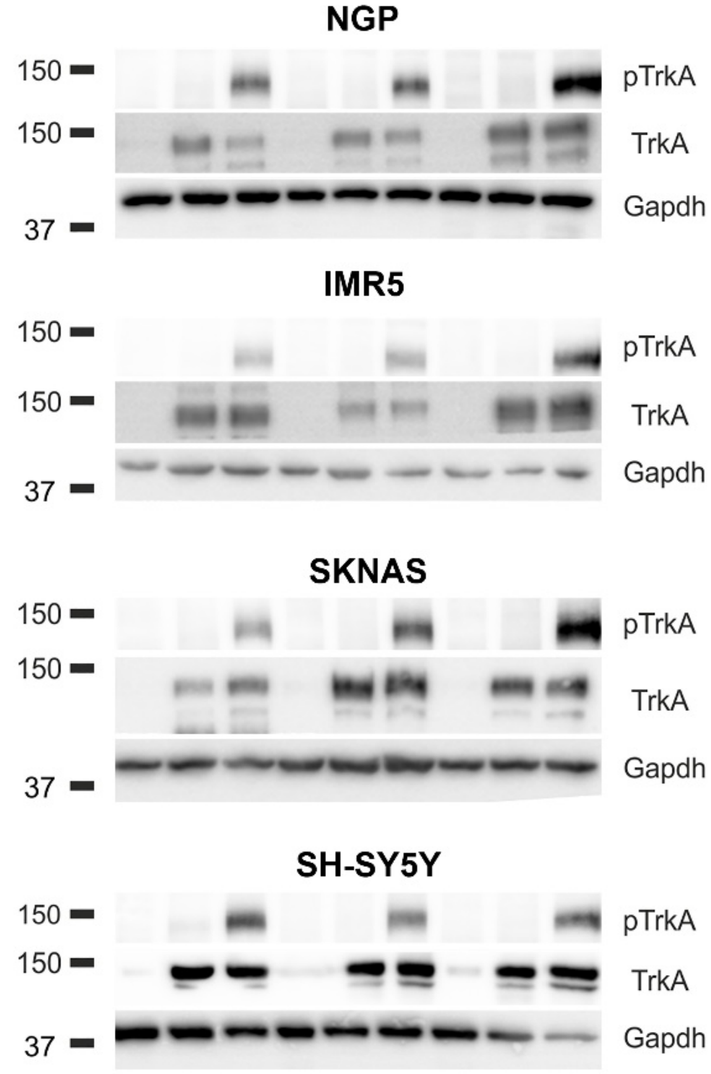

Membranes of the Western blot analyses of the four cell lines NGP, IMR5, SKNAS and SH-SY5Y corresponding to Figure 2B. Names of proteins represent the antibodies which were used on the specific blot fragment for detection of the corresponding protein.

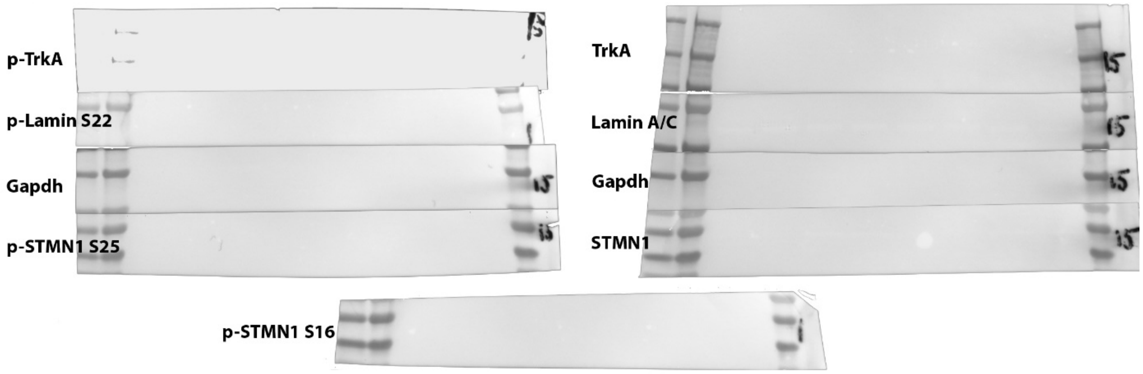

Full membranes of the Western blot analyses of the cell lines IMR5 corresponding to Figure 4D. Names of proteins are given represent the antibodies which were used on the specific blot fragment for detection of the corresponding protein.

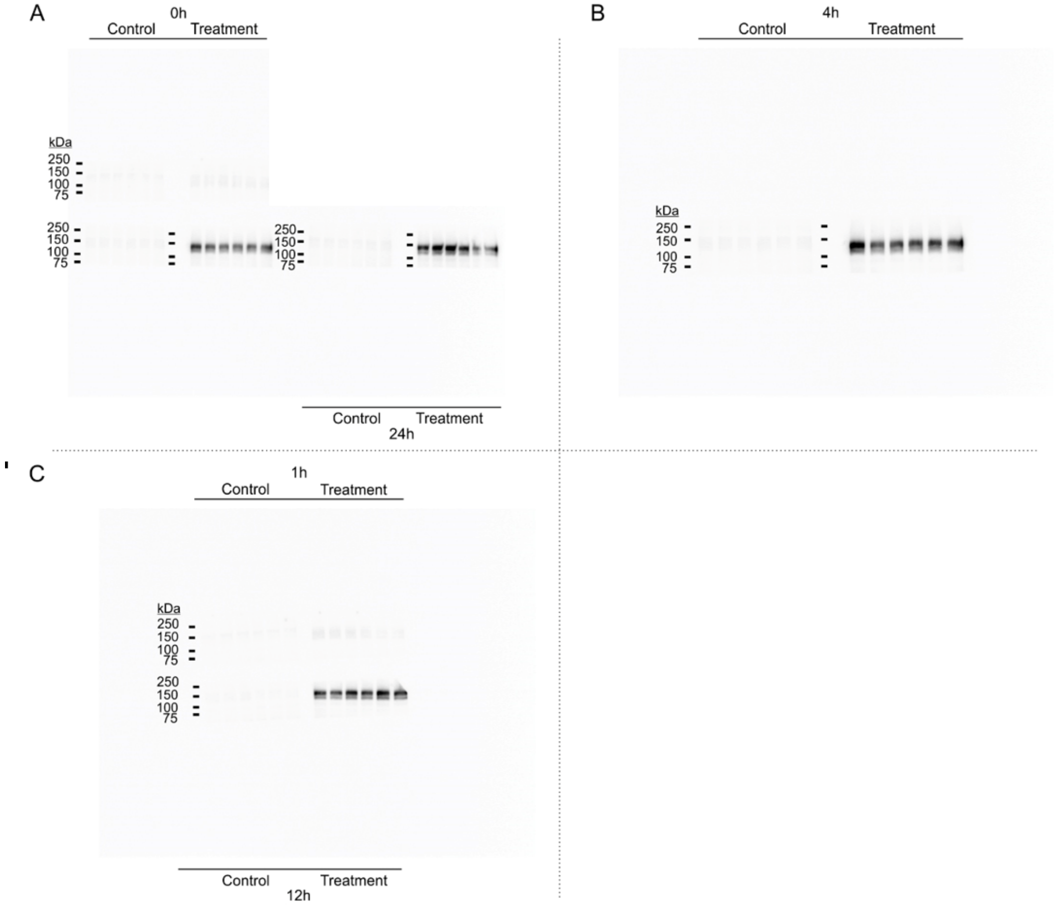

Full membranes fragments of the western blot analyses of NTRK1/TrkA activation shown in Supplementary Figure S1. Six samples per timepoint were initially produced but the first five were used in the subsequent experiments, therefore they were displayed and analyzed. Molecular weight markers were digitally added.

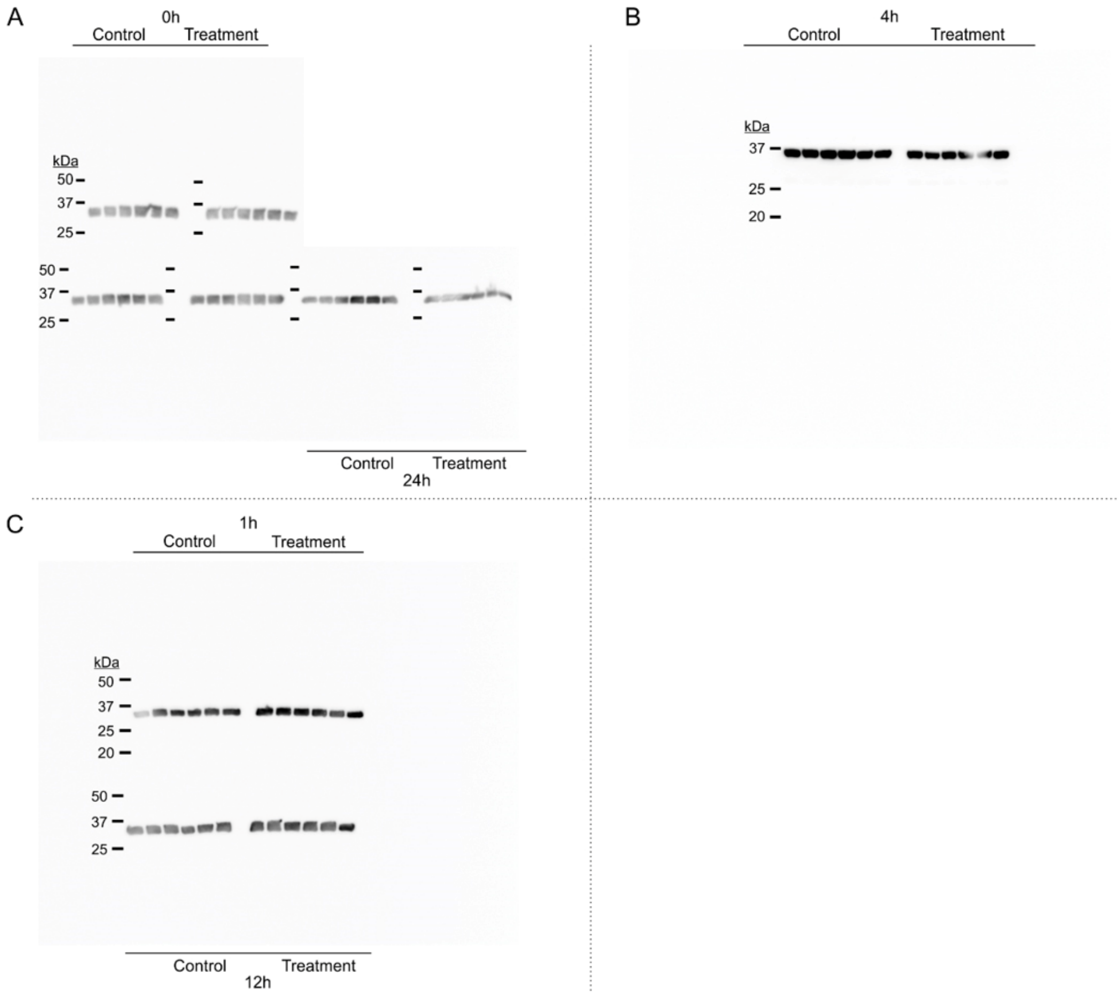

Full membranes fragments of the western blot analyses of Gapdh shown in Supplementary Figure *S*1. Six samples per timepoint were initially produced but the first five were used in the subsequent experiments, therefore they were displayed and analyzed. Molecular weight markers were digitally added.

SY5Y

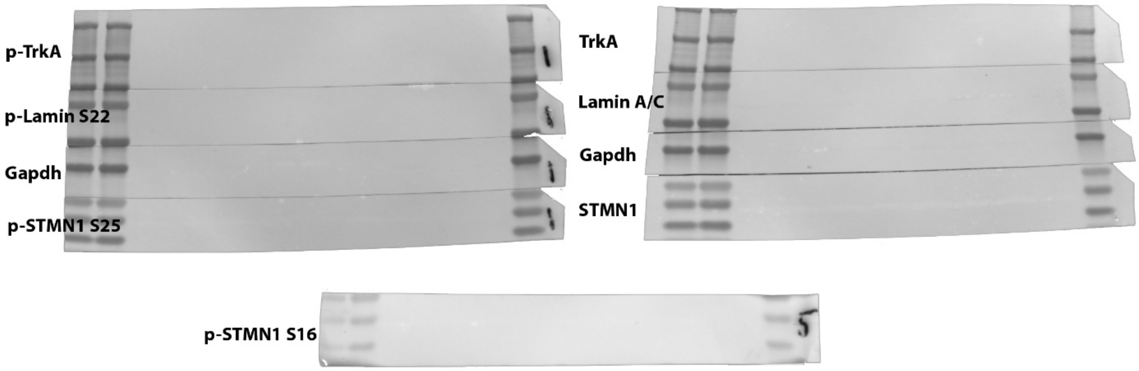

NGP

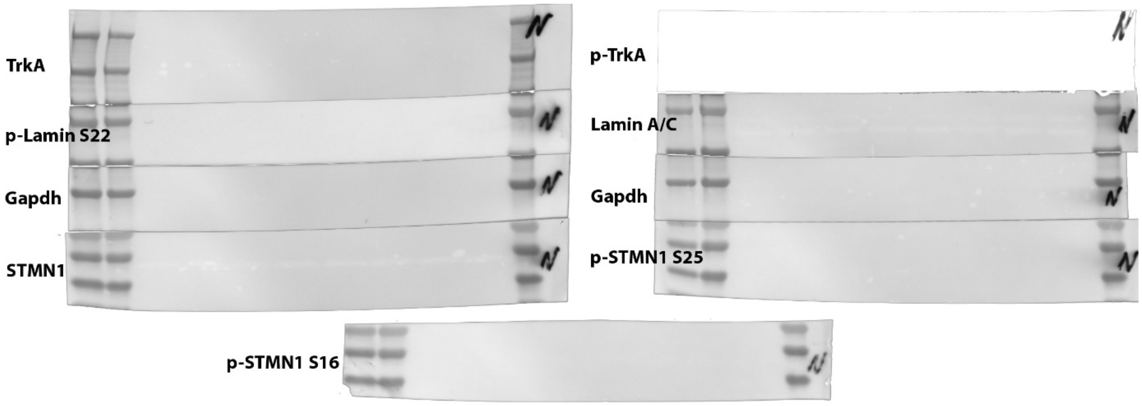

SKNAS

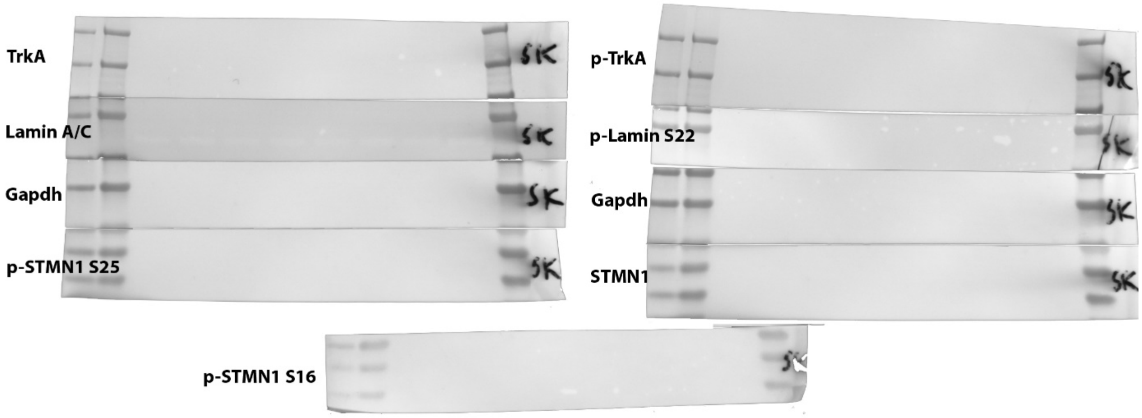

Western blot analyses of the cell lines SY5Y, NGP and SKNAS shown in Supplementary Figure S2. Names of proteins represent the antibodies which were used on the specific blot fragment for detection of the corresponding protein.
