# Supplementary material for: NTRK1/TrkA Signaling in Neuroblastoma Cells Induces Nuclear Reorganization and Intra-Nuclear Aggregation of Lamin A/C
Source: Cancers (Basel). 2021 Oct 21;13(21):5293. doi: 10.3390/cancers13215293 (PMC8582546; doi:10.3390/cancers13215293)
Supplement: Supplementary file 1 [file cancers-13-05293-s001.zip › cancers-1433337-supplementary/cancers-1433337-suppl..pdf]

Supplementary Materials

NTRK1/TrkA signaling in neuroblastoma cells induces nuclear reorganization and intra-nuclear aggregation of Lamin A/C

Lukas Funke, Thilo Bracht, Sebastian Oeck, Karin Schork, Markus Stepath, Sabine Dreesmann, Martin Eisenacher, Barbara Sitek and Alexander Schramm

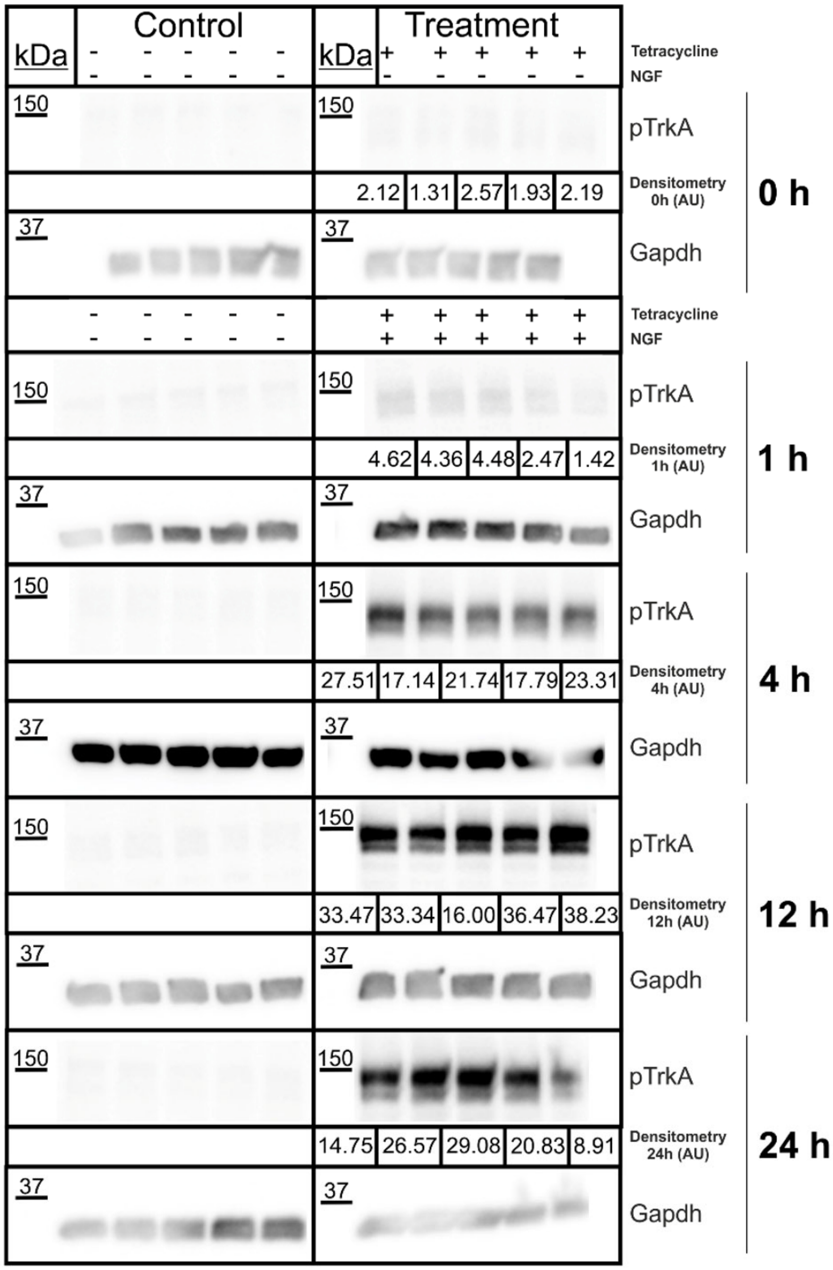

Figure S1. Validation of NTRK1/TrkA expression and activation in IMR5 control and treatment used for subsequent proteomics analyses. NTRK1 activation was validated for all analyzed time points between 0h and 24 h upon treatment with NGF using immunostaining of phosphorylated NTRK1 (pTrkA Y674/675). Ratios between treatment and corresponding control samples were calculated using densitometric measurement results (arbitrary units [AU] displayed below each western blot). Gapdh served as loading control.

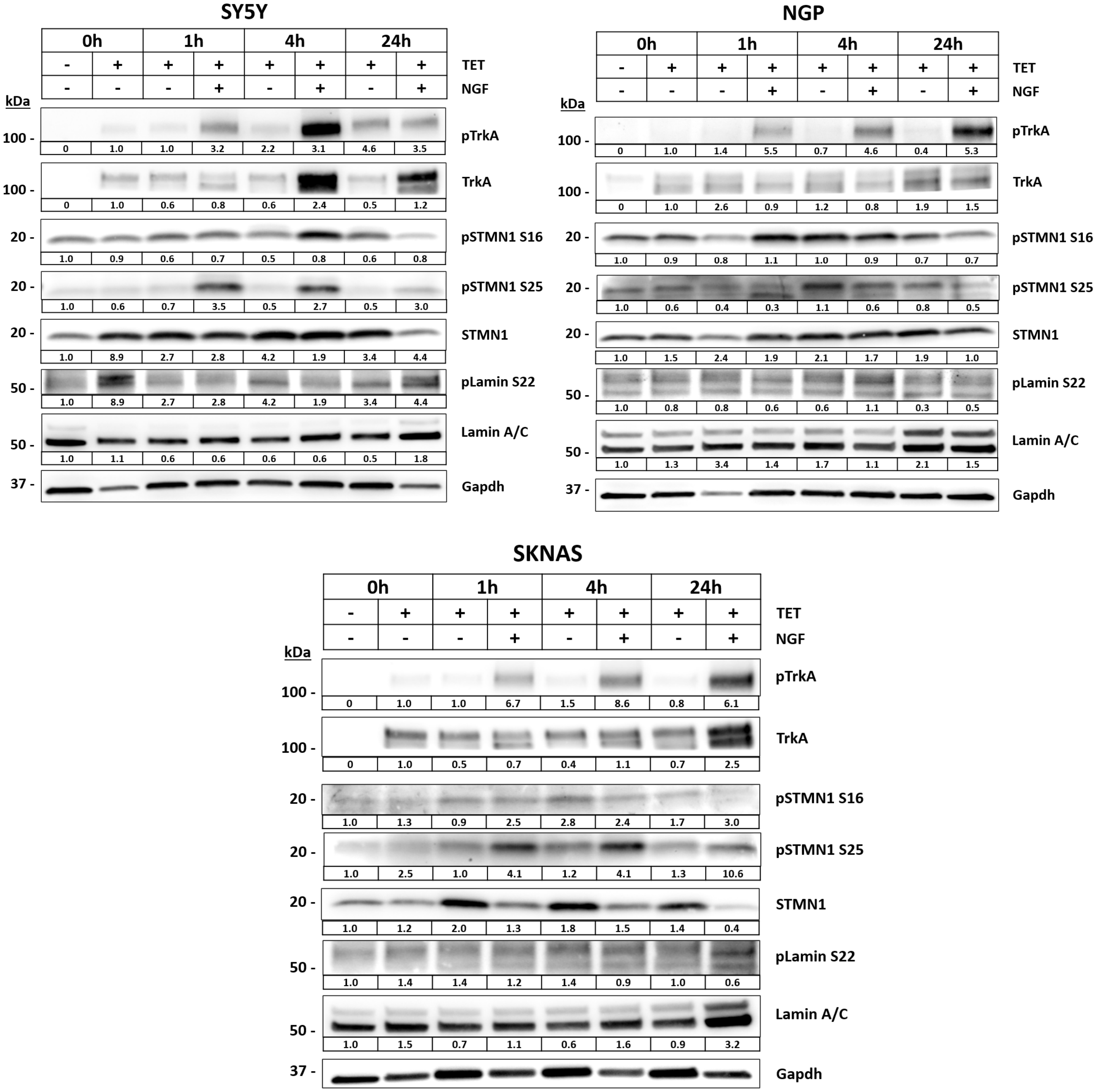

Figure S2. Validation of expression and activation patterns via pLMNA S22, pSTMN1 S16 and S25 in SY5Y, NGP and SKNAS. Ratio between treatment and corresponding control samples were calculated using densitometric measurements. Values are normalised to the respective t = 0h band for each individual antibody.

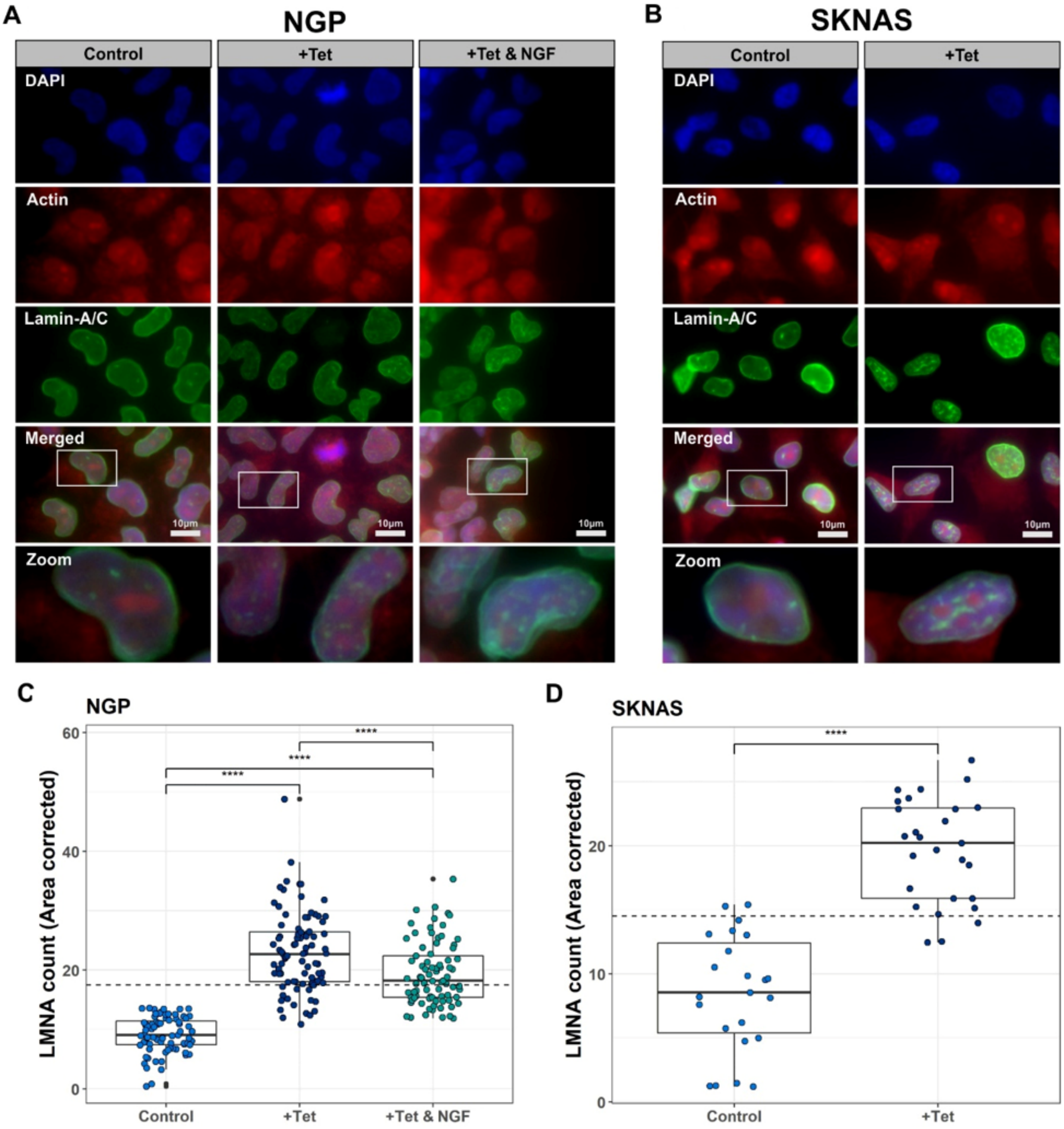

Figure S3. Changes in nuclear localization of LMNA upon NTRK1 expression and activation. Representative immuno*Figure 1.* expression and activation for NGP (A) and SKNAS cells (B) (actin = red, DAPI = blue). Pictures of SKNAS in the +Tet & +NGF-group were excluded due to reduced growth in this experimental setting, resulting in insufficient cell number for staining and statistically sound counting. Area-corrected count of LMNA foci in NGP (C) and SKNAS cells (D). Individual data points correspond to analyzed pictures.
